# Oscillatory Neural Networks Using VO_2_ Based Phase Encoded Logic

**DOI:** 10.3389/fnins.2021.655823

**Published:** 2021-04-16

**Authors:** Juan Núñez, María J. Avedillo, Manuel Jiménez, José M. Quintana, Aida Todri-Sanial, Elisabetta Corti, Siegfried Karg, Bernabé Linares-Barranco

**Affiliations:** ^1^Instituto de Microelectrónica de Sevilla (IMSE-CNM), CSIC and Universidad de Sevilla, Seville, Spain; ^2^Laboratoire d’Informatique, de Robotique et de Microélectronique de Montpellier (LIRMM), University of Montpellier, Montpellier, France; ^3^Department of Science and Technology, IBM Research – Zurich, Rüschlikon, Switzerland

**Keywords:** phase transition materials, VO_2_, nano-oscillators, ONNs, neuromorphics

## Abstract

Nano-oscillators based on phase-transition materials are being explored for the implementation of different non-conventional computing paradigms. In particular, vanadium dioxide (VO_2_) devices are used to design autonomous non-linear oscillators from which oscillatory neural networks (ONNs) can be developed. In this work, we propose a new architecture for ONNs in which sub-harmonic injection locking (SHIL) is exploited to ensure that the phase information encoded in each neuron can only take two values. In this sense, the implementation of ONNs from neurons that inherently encode information with two-phase values has advantages in terms of robustness and tolerance to variability present in VO_2_ devices. Unlike conventional interconnection schemes, in which the sign of the weights is coded in the value of the resistances, in our proposal the negative (positive) weights are coded using static inverting (non-inverting) logic at the output of the oscillator. The operation of the proposed architecture is shown for pattern recognition applications.

## Introduction

Phase-transition materials (PTMs) like vanadium dioxide (VO_2_), with their abrupt switching between states with very different resistivity, are being explored for implementing non-boolean computational paradigms such as neuromorphic architectures. In particular, different groups are exploiting the capability of a PTM device in series with a resistor to oscillate in the implementation of oscillator based computing (OBC).

The field of OBC is not a new idea, with outstanding contributions in the field of logic in the 1950s (von Neumann, 1957; Goto, 1959). In recent years, this idea has received considerable interest and has become an active research area due to the appearance of devices, operating based on very different physical phenomena, with the ability to implement very compact oscillators and with very low energy consumption.

In Csaba and Porod (2020) numerous oscillators are evaluated as potential building blocks of OBC. In terms of energy, PTM-based relaxation oscillators show good performance. They are reported to reduce energy per cycle by more than an order of magnitude when compared to CMOS ring oscillators (ROs). They rank second in terms of energy efficiency, behind only superconducting oscillators.

The most widely used compound as phase-transition material is VO_2_ and the term VO_2_ nano-oscillator has come to be coined (Maffezzoni et al., 2015; Sharma et al., 2015). In addition, they show good performance in terms of scalability and interconnection with electronic circuits, without requiring any conversion between electrical variables and other non-electrical variables as occurs with other nano-oscillators that exploit other physical magnitudes. Numerous experimental results of VO_2_ nano-oscillators have been reported as well as some preliminary results of applications (Shukla et al., 2016; Corti et al., 2018, 2020; Dutta et al.,2019a,b).

Oscillator based computing encompasses a wide variety of operating principles and architectures. In the first place, one can distinguish between those that work with oscillators of ideally identical frequency and the processing corresponds to obtaining a pattern of phase synchronization, phase shift key (PSK) and those that work with oscillators of different frequencies and patterns of frequency synchronization frequency shift key (FSK) (Nikonov et al., 2015).

Oscillator-based-computing phase-shift-key has been applied to obtain solutions to combinatorial optimization problems, difficult to solve in conventional computers. In Wu et al. (2011); Parihar et al. (2017), the problem of graph coloring is solved from the steady-state of a network of oscillators, which represent the nodes, and in which the branches of the corresponding graph are mapped into interconnections that push to separate the phases of the adjacent oscillators. In Dutta et al. (2019a), the resolution of a Max-Cut problem using VO_2_ oscillators is shown experimentally. The comparison with other implementations in terms of scalability, power and quality of the results obtained is very favorable.

Phase shift key has been also applied to explore oscillatory neural networks (ONNs) using a Hopfield-type architecture for associative memories with application in pattern recognition. The neurons in the network are replaced by oscillators and the output is determined by the phase of each one. There are contributions of mathematical analysis with simulations using phase models for neurons (Hoppensteadt and Izhikevich, 1999; Follmann et al., 2015) as well as reporting implementations with different types of oscillators [phase-locked loops and voltage-controlled oscillators (Hoppensteadt and Izhikevich, 2000), non-volatile logic based on magnetic tunnel junctions (Calayir and Pileggi, 2013), micro-electro-mechanical systems and a feedback loop with transconductance amplifiers (Kumar and Mohanty, 2017), comparator and a digital circuit in Jackson et al. (2018), CMOS ring oscillators (Csaba et al., 2016), STOs (Popescu et al., 2018), or VO_2_ (Shukla et al., 2014; Maffezzoni et al., 2015; Corti et al., 2018)]. The implementations based on VO_2_ devices exhibit potential for very low energy computation (Corti et al., 2020). In the case of electrical oscillators, synapses are implemented with resistors or memristors that play the role of weights. In this way, the output of each neuron interacts electrically with the rest. Recently, the potential of ONNs with a small number of neurons to efficiently tackle different image processing tasks has been revealed. Corti et al. (2021) have shown that this approach using VO_2_ oscillators can be exploited for the implementation of commercial high-accuracy image processing architectures based on convolutional neural networks (CNN).

Motivated by the latter type of application, in this paper we describe the implementation of an ONN using VO_2_ based phase encoded logic (PeL). PeL with VO_2_ devices has been recently proposed (Avedillo et al., 2020) by the authors. It uses the phase to encode information in logic circuits and its basic building block is a VO_2_ oscillator which performs a weighted sum of inputs to evaluate its output phase. So we propose to use it to build an ONN. It overcomes some limitations of previously reported VO_2_ ONNs (Corti et al., 2018, 2020).

## Materials and Methods

### Background

#### ONNs With VO_2_ Oscillators

**FIGURE 1:**
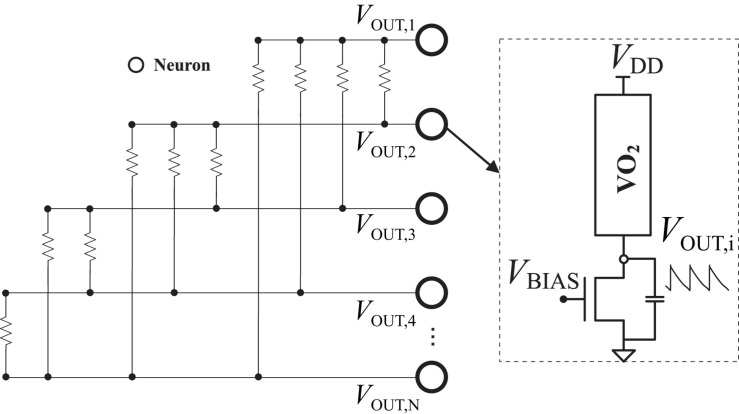
Oscillatory hopfield neural network (OHNN) and VO_2_ oscillator used as neuron.

Figure 1 shows the ONN proposed in Corti et al. (2018, 2020), and the VO_2_ oscillator used as neuron. The resistances implement the synapses among neurons.

**FIGURE 2:**
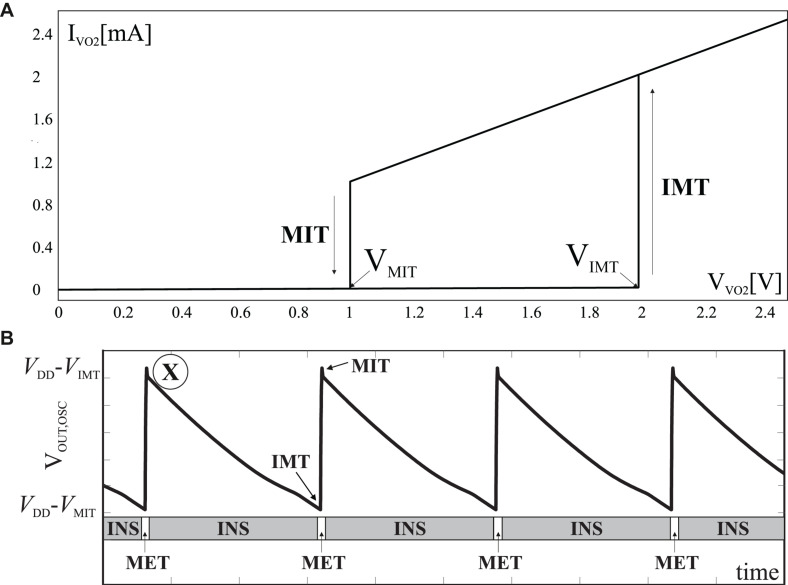
**(A)** I-V curve of the VO_2_ device reported in Corti et al. (2018, 2020). **(B)** Simulated waveform of the output of a VO_2_ oscillator.

Under no electrical stimuli VO_2_ tends to stabilize in the insulating state. When the applied voltage increases and so the current density that flows through it reaches a critical current density, J_*C–IMT*_, insulator to metal transition (IMT) occurs. When the voltage decreases and so the current density reduces below J_*C–MIT*_, the metal to insulator transition (MIT) takes place, transitioning from the metallic to the insulating state. Electrical parameters of its model, are summarized in Table 1. V_*IMT*_ and V_*MIT*_ are the voltages at which the IMT and MIT transition occur, respectively. R_*INS*_ and R_*MET*_ are the resistances in the insulating and metallic state. Since MIT and IMT transitions are abrupt but not instantaneous, transition times (TT_*IMT*_ and TT_*MIT*_) are also included. The I-V characteristic of such device is depicted in Figure 2A. The VO_2_ device has been simulated with a behavioral model as described in Maffezzoni et al. (2015). As expected, in the insulating operating zone the slope is significantly flat, which indicates that the resistance value is very high. On the other hand, in the metallic state, the slope of the I-V curve is clearly steeper, thus implying that the resistance is lower.

**TABLE 1:** Vanadium dioxide (VO_2_) electrical parameters.

V_*IMT*_	V_*MIT*_	R_*MET*_	R_*INS*_	TT
1.99 V	0.99 V	0.99 KΩ	100.2 KΩ	30 ns

Figure 2B depicts simulated waveforms for the oscillator output, V_*OUT,OSC*_, with V_*DD*_ = 2.5 V and V_*BIAS*_ = 2.5 V. The state of the VO_2_ device is also shown to better illustrate the circuit behavior. Regions marked with “INS” label mean that the device is in the insulating state, whereas those marked with “MET” corresponds to the device in the metallic state. When the VO_2_ is in an insulating state (point “X” in Figure 2B), the oscillator output is discharged through the transistor and, therefore, the voltage drop across the VO_2_ (V_*DD*_–V_*OUT,OSC*_) and the current through this device are increased. When the circulating current density reaches the critical value J_*C–IMT*_, the VO_2_ switches to the metallic state. On the other hand, the switching to the metallic state occurs once the VO_2_ voltage reaches V_*IMT*_, when the output is then charged through the VO_2_ device. Due to the low R_*MET*_ value, this charging is very fast and leads to a reduction of the voltage seen by the VO_2_ until it reaches V_*MIT*_ and the MIT occurs. Finally, note that the voltage V_*BIAS*_ can be used to control the frequency of the signal.

This ONN works as an associative memory with application in pattern recognition. The ONN state is defined by the phase of each neuron. There are states which are stable and others which if entered converge to a stable one. For pattern recognition, a set of patterns (called training patterns) are said to be stored in the network, which means the network is configured so that the state corresponding to such patterns are stable. When the network is placed in a state corresponding to a distorted version of a training pattern, it evolves to a training pattern, ideally to the most similar one. Placing the ONN in a given state means fixing a specific phase for each oscillator. This is achieved by suitably delaying the switching on of the supply voltage of each oscillator.

The stable states of the network are determined by the resistance values connecting the oscillators, which plays the role of the weights of the neural network. The required weights to store a given set of training patterns are derived applying the well-known Hebbian rule (Hebb, 1949) and then mapped to resistance values.

There are several challenges in the operation of this ONN. First, in order to work properly, the neurons must all synchronize in frequency. Although ideally all neurons are identical, and so they oscillate at the same frequency, in practice this is not the case because of different reasons. Variability between the VO_2_ devices is the main one. Secondly, both positive and negative values must be mapped to resistance values. Note that positive weights mean that the phase of both associated oscillators should be pulled to each other while negative ones should have the contrary effect. Although, there are results showing that two oscillators coupled with enough large resistance value end in anti-phase configuration, the device-to-device of variability can have a great impact on this behavior, especially when many oscillators are coupled. The ONN we propose aims at addressing these challenges.

#### VO_2_-Based PeL Description

Key components of PeL logic are VO_2_ oscillators with only two possible phases, 180° apart. The oscillating phase depends on the phases (also discretized) of the applied inputs. That is, the resulting phase is a logic function of the inputs. In particular, it implements a majority functionality. The output phase is the majority phase.

**FIGURE 3:**
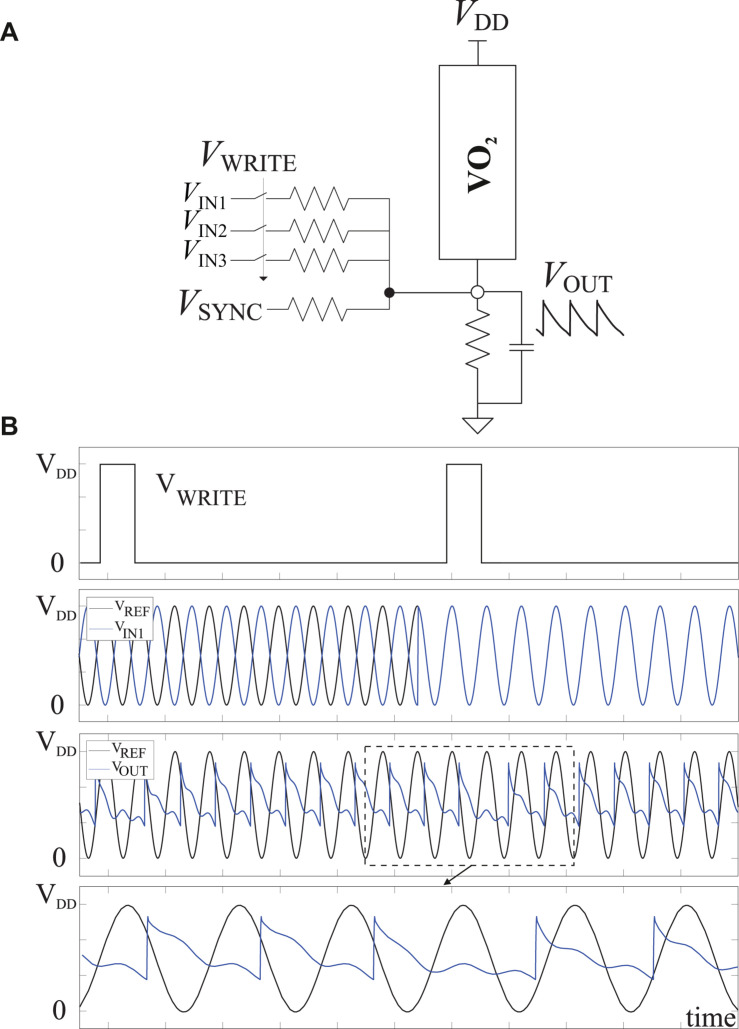
PeL three-input majority gate. **(A)** Schematic. **(B)** Waveforms.

Figure 3A depicts the schematic of a three-input majority gate (Avedillo et al., 2020). It exploits sub harmonic injection locking (SHIL) to stabilize the oscillator frequency against variability effects and to discretize its phase. This is achieved by injecting a synchronization signal (V_*SYNC*_), which ranges between 0 and V_*DD*_, of frequency close to twice the natural frequency of the oscillator. The oscillator outputs exhibit half such injected frequency. The phases of the input signal are represented in terms of that of a reference signal (V_*REF*_) external to the oscillator.

When signal V_*WRITE*_ activates, the oscillator phase is forced to that of the majority phase of its three inputs. For example, for (V_*IN1*_, V_*IN2*_, V_*IN3*_) = ($\bar{{\text{V}}_{\text{REF}}}$, $\bar{{\text{V}}_{\text{REF}}}$, V_*REF*_), the phase corresponding to $\bar{{\text{V}}_{\text{REF}}}$ is stored and for (V_*IN1*_, V_*IN2*_, V_*IN3*_) = (V_*REF*_, $\bar{{\text{V}}_{\text{REF}}}$, V_*REF*_) the phase corresponding to V_*REF*_ is stored. Figure 3B depicts simulation results for those input combinations. From top to bottom the V_*WRITE*_, V_*IN1*_ and the oscillator output, V_*OUT*_, are shown. V_*IN1*_ is the only changing input and determines the output value. A reference signal (V_*REF*_) is also displayed to ease identification of the phase of each signal. That is, V_*IN1*_ is $\bar{{\text{V}}_{\text{REF}}}$ initially and then changes to V_*REF*_. Note the phase change of V_*OUT*_ in response to the application of the second V_*WRITE*_ pulse.

### PeL-Based ONN Architecture

#### The New Neuron

**FIGURE 4:**
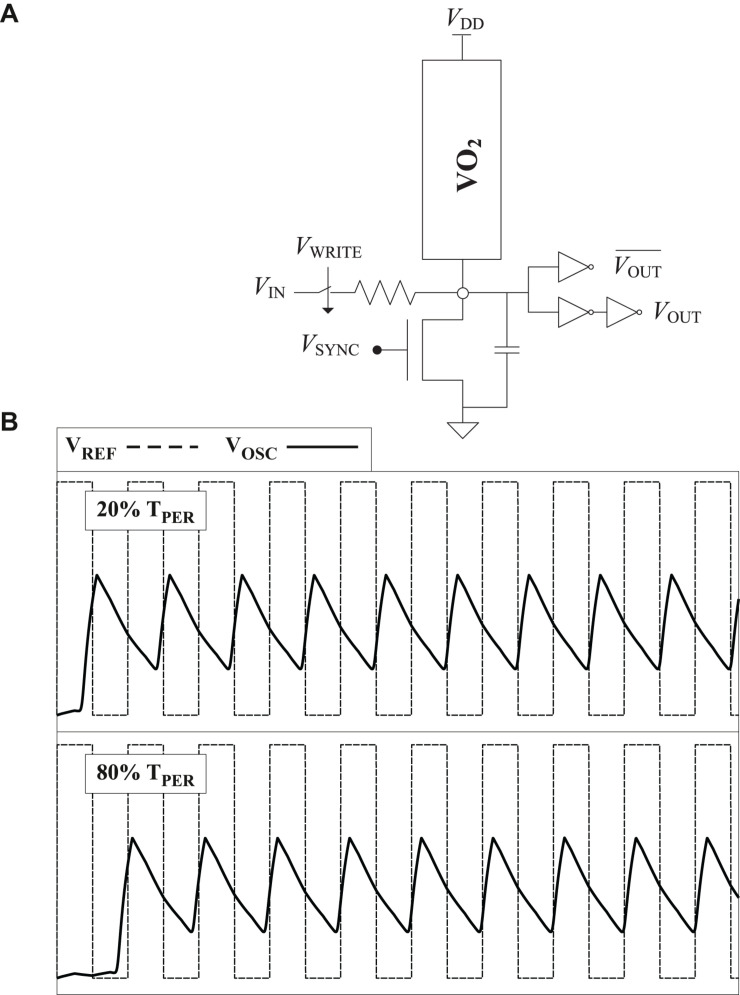
Proposed neuron. **(A)** Schematic. **(B)** Operation for two values of the delay of the supply voltage.

Figure 4A depicts the topology proposed for the neuron Note the use of the synchronization signal V_*SYNC*_, like in PeL, although it is injected through the gate of the transistor avoiding the injection resistor. In the neuron, it contributes to reducing period variations due to device variability, which translates to frequency synchronization advantages in the ONN network. Figure 4B depicts its operation. The oscillator output before the static logic, V_*OSC*_, and a reference signal are shown. The use of SHIL reduces the number of phases to two. It can be observed that the oscillation phase can be controlled by the supply voltage delay like in the original ONN described in the background section. Supply voltages delays under half the oscillation period (top waveform in Figure 4B) lead to one phase and delays over half period (bottom waveform) force the other phase.

**FIGURE 5:**
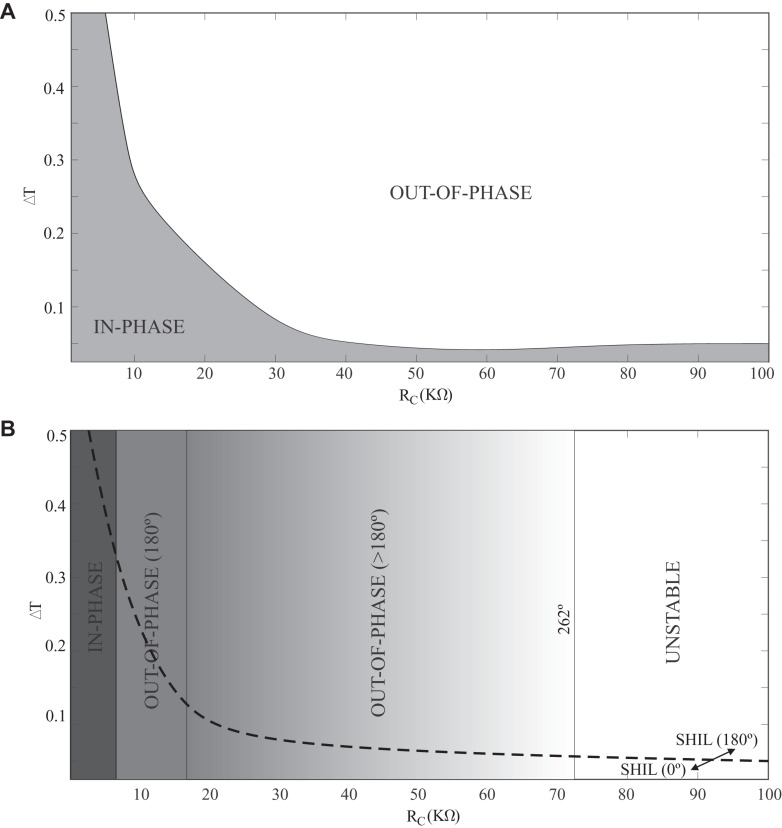
ΔT vs. R_C_ plot for in-phase/out-of-phase operation of two conventional coupled oscillators. **(A)** Nominal scenario. **(B)** Considering R_INS_ and R_MET_ variations. The boundary for the in-phase/out-of-phase operation of two SHIL-based oscillators is depicted with a dashed line.

It is interesting to study the robustness of the ONNs against variations in the electrical parameters of the VO_2_ (resistance in the insulating and metallic state and switching voltages between states). In this sense, conventional implementations of ONNs are sensitive to these variations. Figure 5A depicts a design space for the phase difference between two identical coupled oscillators in which the variables are the time difference between oscillator initialization (ΔT) and the coupling resistance (R_*C*_). Note how two clearly differentiated regions corresponding to an in-phase (0°) and out-of-phase (180°) operation are observed. Figure 5B reproduces the previous plot by considering variations of 10% of the insulating and metallic resistances of the VO_2_ device of one of the oscillators with respect to the nominal scenario (R_*INS,1*_ = 0.9⋅R_*INS,2*_ and R_*MET,1*_ = 0.9⋅R_*MET,2*_). Significant differences are observed given that there are phase differences other than 180° for the out-of-phase region and even an area of unstable behavior. Unstable behavior means that both oscillators are not able to synchronize. Also, note that in the plot corresponding to ideal oscillators there are resistance values for which both in-phase and out-of-phase are possible depending on the initial delay (phase difference between) the two oscillators. This bistability, which is interesting from the point of view of the ONN functionality, is not observed in the plot with variability (Figure 5B). In this figure, the boundary between the in-phase and out-of-phase operating regions for two coupled oscillators using SHIL, and considering the same variation between the VO_2_ resistances, has been represented using a dashed line. Two clearly distinct regions of operation are observed like in the ideal plot. These results reveal that SHIL has significant benefits in that inherently two complementary phases are obtained at the output and this is more tolerant to variations in the parameters of VO_2_.

#### Synapse

**FIGURE 6:**
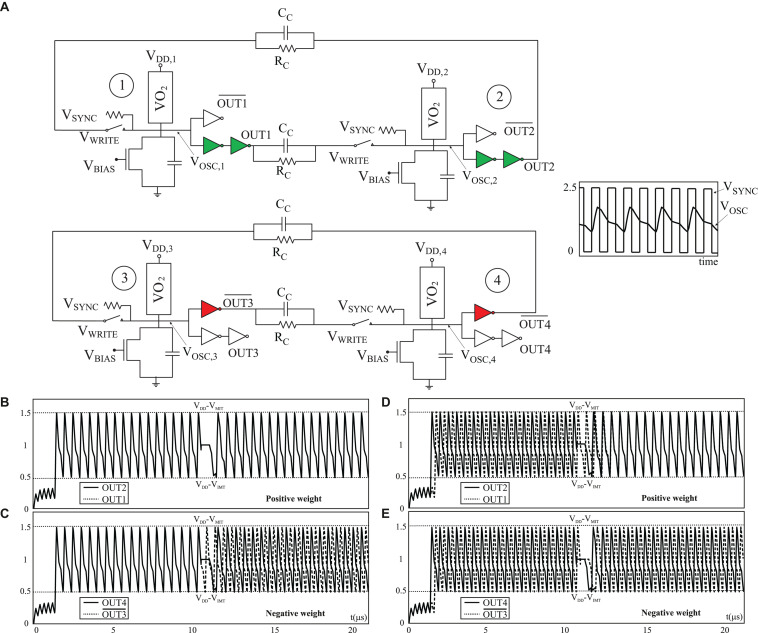
**(A)** Schematics of the interconnection of two neurons with a positive (green) and a negative (red) weight. **(B–E)** Waveforms showing the operation of both interconnection schemes.

The proposed interconnection scheme encodes the sign of the weights in the way the neurons are connected unlike the original ONN, which relies just on resistance values. Figure 6A shows two possible scenarios for the interconnection of two neurons using positive and negative weights. When interconnecting using positive weights the output of each is connected to a buffer (marked in green), while for encoding negative weights an inverter (marked in red) is used. Note that unlike the original ONN, which uses a bidirectional interconnection mechanism with a single coupling resistor between two neurons, in the proposed scheme the interconnection is unidirectional and therefore two coupling resistors must be used. The rationale behind using the oscillation signal but complemented for negative weights is that in an ONN, a negative weight must push away the phases of the two neurons which is equivalent to pull the phase of the neurons to the complement of the other one. The buffer is used for positive weights so that the shape of the two outputs of the neuron are similar. Figure 6 also shows simulation results of both interconnection schemes. The four scenarios are illustrated. Initially in-phase neurons connected with positive coupling weight (Figure 6B) and with negative weight (Figure 6C) and initially out-of-phase neurons connecting with positive (Figure 6D) and negative (Figure 6E) weights. Note neurons coupled with positive weights end up in phase independently of their initial states (Figures 6B,D). Similarly, neurons coupled with negative weights evolve toward out of phase (Figures 6C,E).

#### Network Operation

The switch at the input of the neurons allows disconnecting the coupling among them by fixing the V_*WRITE*_ signal to a low voltage. This is used at the beginning of the operation to initialize the ONN state (phase of each neuron oscillation). As mentioned before, this is the way an input pattern is applied to the network. After application of successively positive V_*WRITE*_ pulses enable interaction among the neurons and the network state evolves toward ideally the closest stored pattern.

## Results

### PeL Associative Memory

**FIGURE 7:**
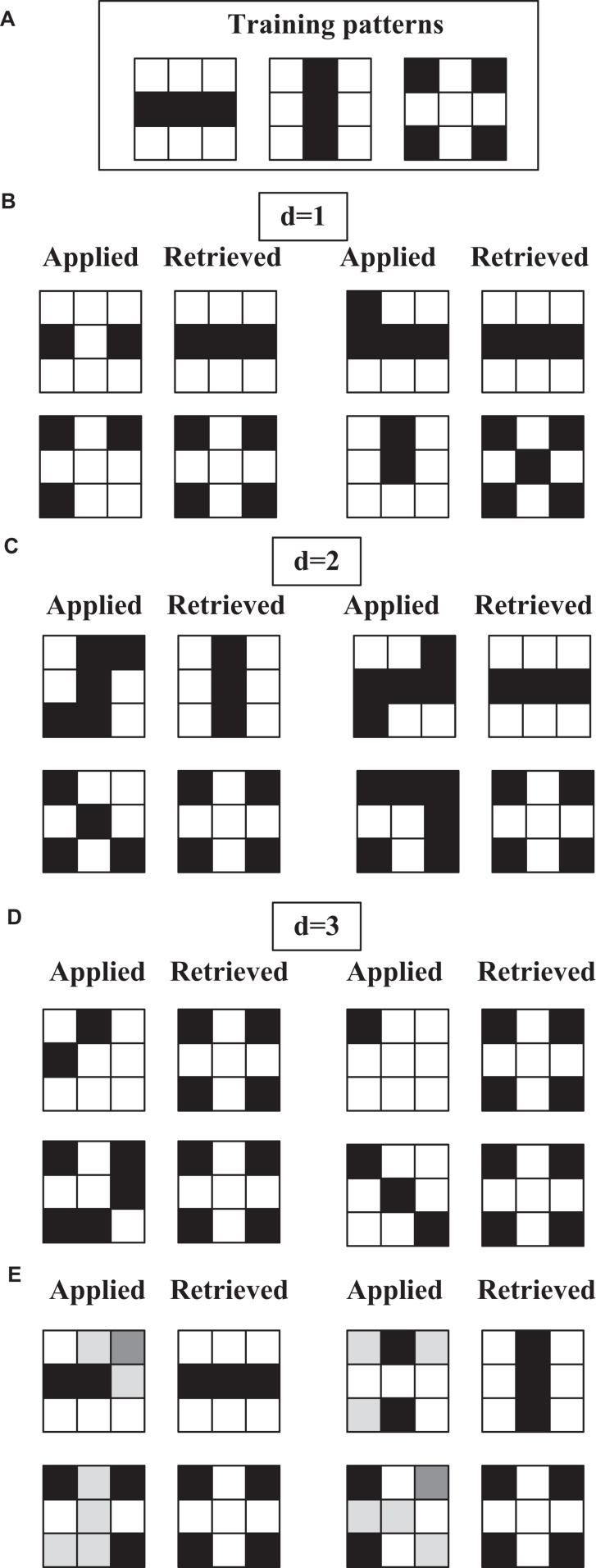
3 × 3 ONN experiment. **(A)** Training patterns. **(B–D)** Applied and retrieved patterns for Hamming distances of 1, 2, and 3, respectively, from the training patterns. **(E)** Applied and retrieved patterns for test patterns generated from the training ones adding gray pixels with different intensities.

As a first experiment to verify the operation of the network, we propose the training and test patterns corresponding to 3 × 3 pixel size images shown in Figure 7. In this demonstration (and the following ones), the supply voltage is 2.5 V, the oscillator capacitance is 100 pF and the coupling capacitance is 0.05 pF. The obtained Hebbian weight matrix exhibits two positive and two negative values which have been mapped to resistances, 100 KΩ and 300 KΩ, respectively.

**FIGURE 8:**
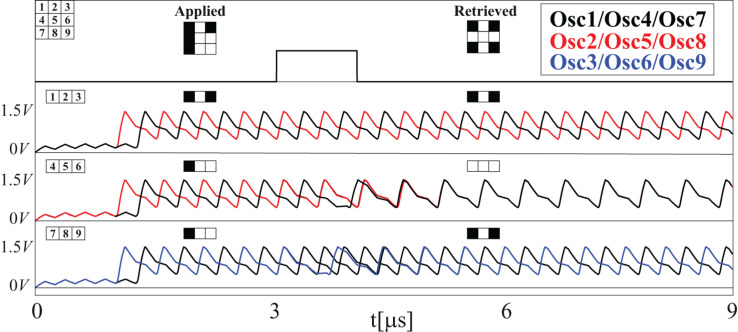
Waveforms corresponding to one of the experiments illustrated in Figure 7.

The results are shown in Figures 7B–E. Waveforms corresponding to one of these experiments are shown in Figure 8. Specifically, the write pulse and the outputs of the oscillators before the inverter/buffer are shown. Note that the outputs have been grouped for each of the rows. After the first writing cycle, the outputs of bits 4 and 9 change their phase and, thus, the expected training pattern is recovered.

Coming back to Figure 7, the test patterns for which results are shown have been categorized into four groups in order to facilitate the analysis. The first three groups represent test patterns at Hamming distances of 1 (Figure 7B), 2 (Figure 7C), and 3 (Figure 7D) from the training or stored patterns. To consider a result as correct, the retrieved pattern must match the training pattern that has the lowest Hamming distance with respect to the test pattern. All but one test pattern are correctly retrieved. In fact, the one that has not been recovered, the vertical line with a missed pixel in Figure 7B, is at distance of 1. However, in order to fairly analyze the pattern recognition performance of the proposed ONN, it is important to be aware of the capabilities of the Hopfield model itself. It is well known that even the ideal model is not able to correctly retrieved any number of stored patterns, but its capacity depends on the number of neurons, the correlation among the patterns to be stored, and the learning rule. In the case of random training patterns, the maximum number which can be reliably stored (P_*ERROR*_ < 1/N) is 0.14⋅N for the Hebbian learning rule (Hopfield, 1982). That is, we should not expect perfect retrieval since we are storing too many patterns for the network size. So, it is interesting to investigate also the performance of the Hopfield network on this example. For that, the same example has been simulated with a MATLAB model of a Hopfield network. Its accuracy is under 80.5% and in particular, neither it recovers the vertical line from the one without the bottom pixel. From our simulations, we have also observed that the third training pattern is easier to be retrieved and we have confirmed that this is also the case for the model. This is in agreement with this pattern being the one exhibiting the smaller energy minimum and so exercising stronger attraction.

The last group (Figure 7E) depicts test patterns generated from the training ones adding gray pixels with different intensities. As explained before, gray values in the input image are encoded in distinct initialization times of the oscillators. It can be observed that the most similar training pattern is retrieved in all cases.

### PeL ONN for Character Recognition

**FIGURE 9:**
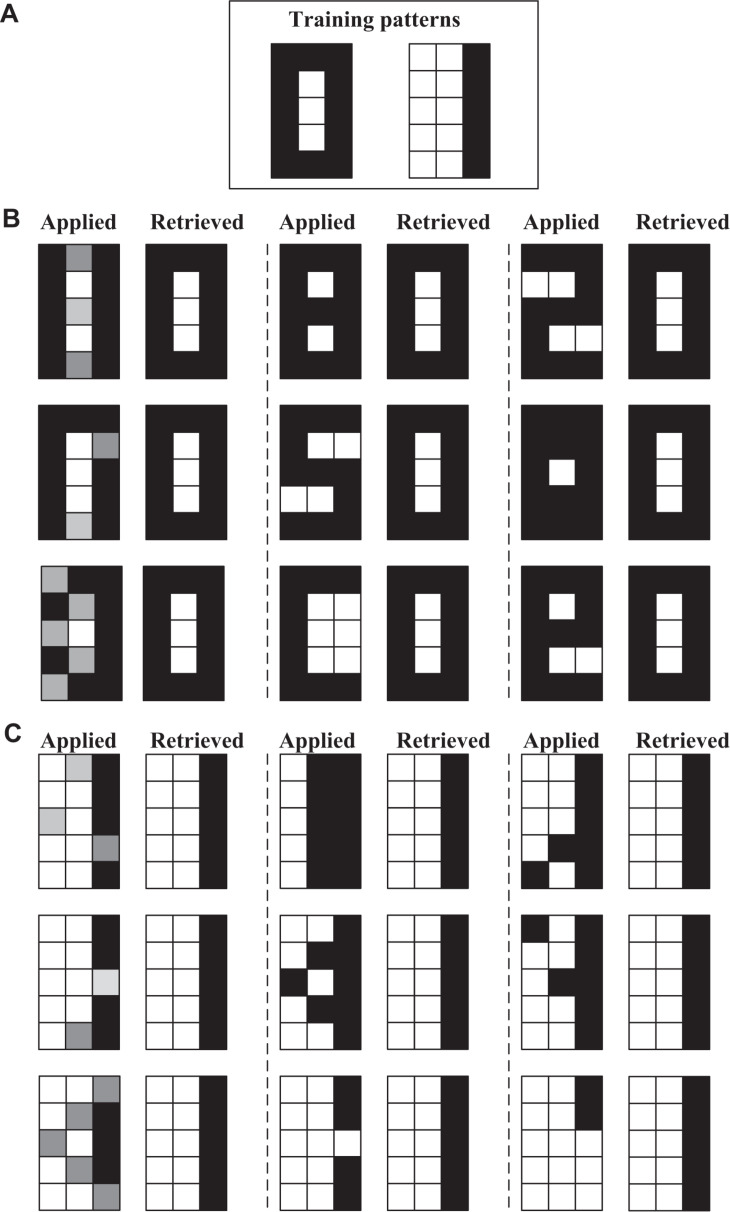
5 × 3 ONN experiment. **(A)** Training patterns. **(B,C)** Applied and retrieved patterns corresponding to “0” and “1,” respectively.

In order to further illustrate our proposal, we have designed an ONN for character recognition. For this, using the Hebbian rule, weights have been derived to store 5 × 3 pixels representations of digits “0” and “1” as shown in Figure 9A. These weights have been mapped to resistance values (R_*C*_ = 200 KΩ and R_*C*_ = 400 KΩ for strong and weak coupling weights, respectively) and an ONN with our architecture has been simulated with them using HSpice electrical simulator.

The performance of ONN has been evaluated using the set of 18 test patterns shown in Figure 9. These experiments have been grouped in sets of nine corresponding to an expected output of the image “0” (Figure 9B) and “1” (Figure 9C) based on the criterion of minimum Hamming distance, in which both the applied and retrieved patterns are shown. Within each set of 9, the first column represents noisy versions of the corresponding training pattern. The other two columns represent harder test patterns in which some bits have been completely flipped. The results show that all applied patterns were successfully retrieved.

## Discussion

A novel ONN architecture based on phase encoding is proposed and its operation as associative memory is shown. Phase information storage using oscillators with VO_2_ devices and subharmonic injection locking is exploited for the neurons. SHIL has shown to greatly increase the variability robustness with respect to free VO_2_ oscillators. The proposed mechanism of interconnecting neurons encodes the sign of the weight by using static logic to force a phase change instead of just having different resistance values. This architecture allows overcoming some of the challenges that arise in other implementations of VO_2_-based ONN, including improved robustness against variability and simplifying mapping of weights to resistance values.

## Data Availability Statement

The raw data supporting the conclusions of this article will be made available by the authors, without undue reservation.

## Author Contributions

JN, MA, and JQ developed the main concepts. JN performed all the simulations. All authors assisted in the writing of the manuscript and developing the concepts.

## Conflict of Interest

EC and SK were employed by the company IBM. The remaining authors declare that the research was conducted in the absence of any commercial or financial relationships that could be construed as a potential conflict of interest.
